# Hybrid-Driven Origami Gripper with Variable Stiffness and Finger Length

**DOI:** 10.34133/cbsystems.0103

**Published:** 2024-04-09

**Authors:** Zhuang Zhang, Weicheng Fan, Yongzhou Long, Jiabei Dai, Junjie Luo, Shujie Tang, Qiujie Lu, Xinran Wang, Hao Wang, Genliang Chen

**Affiliations:** ^1^ State Key Laboratory of Mechanical System and Vibration, and Shanghai Key Laboratory of Digital Manufacture for Thin-Walled Structures, Shanghai Jiao Tong University, Shanghai, 200240, China.; ^2^ School of Engineering, Westlake University, Hangzhou, Zhejiang, 310030, China.; ^3^ Academy for Engineering and Technology, Fudan University, 200433, Shanghai, China.; ^4^ Reds Lab, Dyson School of Design Engineering, Imperial College London, London, SW7 2DB, U.K.; ^5^ META Robotics Institute, Shanghai Jiao Tong University, Shanghai, 200240, China.

## Abstract

Soft grippers due to their highly compliant material and self-adaptive structures attract more attention to safe and versatile grasping tasks compared to traditional rigid grippers. However, those flexible characteristics limit the strength and the manipulation capacity of soft grippers. In this paper, we introduce a hybrid-driven gripper design utilizing origami finger structures, to offer adjustable finger stiffness and variable grasping range. This gripper is actuated via pneumatic and cables, which allows the origami structure to be controlled precisely for contraction and extension, thus achieving different finger lengths and stiffness by adjusting the cable lengths and the input pressure. A kinematic model of the origami finger is further developed, enabling precise control of its bending angle for effective grasping of diverse objects and facilitating in-hand manipulation. Our proposed design method enriches the field of soft grippers, offering a simple yet effective approach to achieve safe, powerful, and highly adaptive grasping and in-hand manipulation capabilities.

## Introduction

Drawing inspiration from the inherent compliance and softness of biological systems, there’s a burgeoning interest in soft robots and actuators, primarily for their promising capabilities in human–robot interaction and complex environments [1,2]. Unlike traditional rigid actuators, their soft counterparts are crafted from hyperelastic materials and possess infinite degrees of freedom (DoF). Such inherent flexibility offers enhanced safety and sensitivity independent of the control algorithm [3,4], with the potential showcased in diverse areas like locomotion, manipulation, and wearable and biomedical devices [5–12]. Even though actuation techniques such as thermal, optical, magnetic, and electrical have been introduced [13–16], soft pneumatic actuators (SPAs) continue to gain traction for their affordability, straightforward actuation, user safety, and uncomplicated creation process. Typically, SPAs mirror cephalopods in design, lacking an endoskeleton and predominantly made from elastomers like silicone rubbers [1,17]. When air pressure is applied via inbuilt channels, these hyperelastic designs can bend, twist, and extend/contract [18–20]. This kind of motion induced by deformation is both versatile and interaction-safe, making SPAs (particularly those with a bending motion) ideal for crafting a range of soft grippers [21–24]. Nevertheless, the performance of these soft grippers deteriorates when a high payload is required, owing to the stretchable, isotropic mechanical characteristics of SPAs. Moreover, the existing SPAs generally possess fixed workspaces that depend on their predesigned parameters and are limited by their stretchabilities, making the corresponding grippers possess a fixed grasping range for limited objects [25,26].

A variety of soft pneumatic grippers have been developed and can be divided into several categories according to their constructions. Pneu-net structure-based [27,28] and fiber-reinforced grippers [29,30] are the most classic designs with prominent intrinsic compliance and output deformation. On the basis of these designs, the integration of phase-changing materials [31,32] and jamming elements [33,34] further lead to readily achievable variable stiffness, since the finger stiffness depends on these integrated materials due to the relatively lower modulus of the silicone substrates. The load-carrying capabilities of these soft designs can be thus enhanced under higher stiffness values. In addition to these actively controlled stiffness, soft–rigid hybrid designs, both inner rigid [35,36] and outer rigid [37,38], also result in stronger structures and better load-carrying capabilities because of the reduced passive deformation of soft chambers in undesirable directions. All these design optimizations can effectively enhance the load-carrying capacity of soft pneumatic grippers. However, these grippers still process fixed finger lengths, rendering each gripper to be able to only grasp objects of a certain size.

**Fig. 1.**
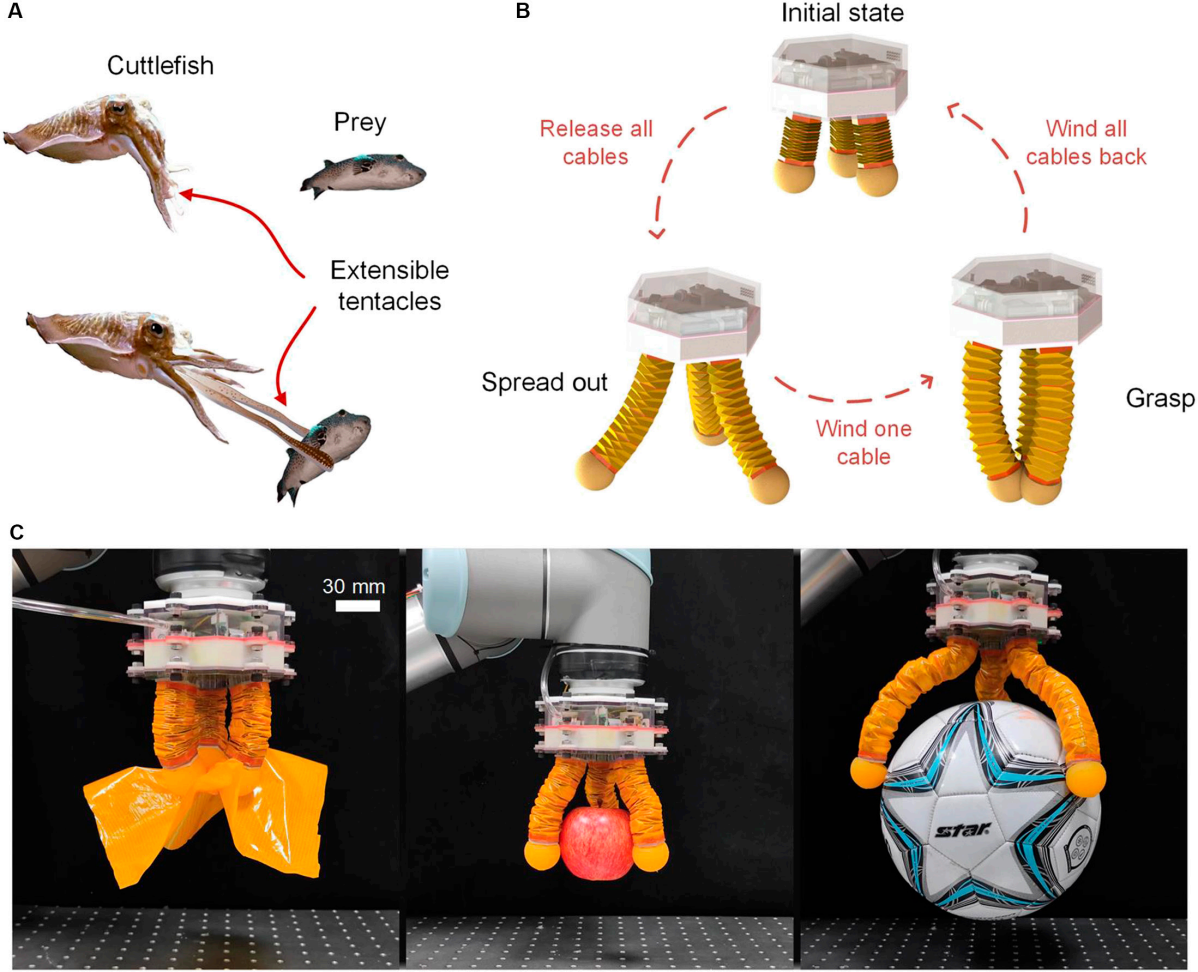
Bioinspired grasping of the origami gripper. (A) Predating process of a cuttlefish. Instead of getting closer, the cuttlefish elongates its tentacles and enlarges its workspace to grab its prey. (B) Bioinspired gripper with a soft–rigid hybrid origami structure. The origami chamber can be readily compressed and elongated under the hybrid actuation of cables and air pressure, resulting in tunable finger length. (C) The grasping performance of a gripper prototype, ranging from a 0.1-mm-thick fabric piece to a 215-mm-diameter football, under different finger lengths.

In nature, not all creature organs have fixed sizes. One interesting example is the tentacle of cuttlefish (Fig. 1A). Unlike octopus, the cuttlefish has 4 pairs of arms with fixed lengths and one pair of tentacles that can freely extend/contract [39]. These soft tentacles can generate high-range and powerful motion, making long-distance predation possible. As for soft grippers, these 2 characteristics are still of high significance. The tunable effective length can enlarge the grasping of the gripper and offers the potential to select the most suitable size according to the interacted object; the powerful actuation can make soft fingers capable of holding heavier objects, enhancing their practical value. Nevertheless, unlike the numerous existing designs of soft grippers with variable stiffness to carry higher loads, few gripper designs exhibit tunable finger lengths. Yoon et al. [40] adopted a tendon-driven length-adjustable linkage mechanism in the underactuated finger, making the resulting gripper able to elongate the fingers for an increased task space or shorten them for a finer spatial resolution. However, this gripper possesses a rigid structure, performing less compliance and a low tuning range of finger length. Our previous work proposed a lightweight but powerful soft gripper based on fabric substrates, whose finger length can be substantially changed through winding the fabric chambers [41]. However, the deformation of the fabric chamber is constant upon pressure, resulting in an uncontrollable grasping process under a specific finger length. Benefiting from their reconfigurable features and unique mechanical properties, origami structures [42–46] have been widely adopted in building versatile robots in recent years [17,47–50]. The high folding/unfolding ratio of the origami makes it suitable to be utilized to construct length-tunable robotic fingers. Nevertheless, few attempts have been made [51], and the effectiveness of tuning the finger lengths on grasping and in-hand manipulation performances has yet to be demonstrated.

Here, inspired by the functionalities of cuttlefish tentacles, we propose a design strategy to make soft grippers simultaneously perform variable stiffness and finger lengths, based on pneumatic/cable-driven hybrid origami chambers. Unlike the molding method of conventional silicone-based gripper fingers, the proposed fingers are constructed with thermoplastic urethanes (TPU)-coated fabric and discrete thin metal sheets, forming soft–rigid hybrid origami chamber structures with a high contraction/extension ratio. Three independently controlled cables are integrated into each chamber to confine the motion of the origami chamber, rendering tunable finger lengths when synchronously pulling the 3 cables and freely inward or outward bending when differentially pulling the 3 cables (Fig. 1B). The positive pneumatic pressure is utilized to inflate the origami chamber, keep the cables in tension, and form an antagonistic actuation system with the cables, making the finger stiffness tunable via active controlling of the input pressure value. Such a combination leads to a soft gripper with a high grasping range, capable of grasping from a 0.1-mm-thick fabric piece to a 215-mm-diameter football (Fig. 1C). Moreover, the kinematic model of the hybrid-driven soft finger is also established to precisely control the bending and the tip position of the finger, enabling the in-hand manipulation that is conventionally performed by rigid robotic hands and grippers also achievable by the soft counterparts. The proposed design method can enrich the field of soft grippers in a simple manner to realize high-range and highly controllable grasping.

## Materials and Methods

### Design and working principle of the gripper

**Fig. 2.**
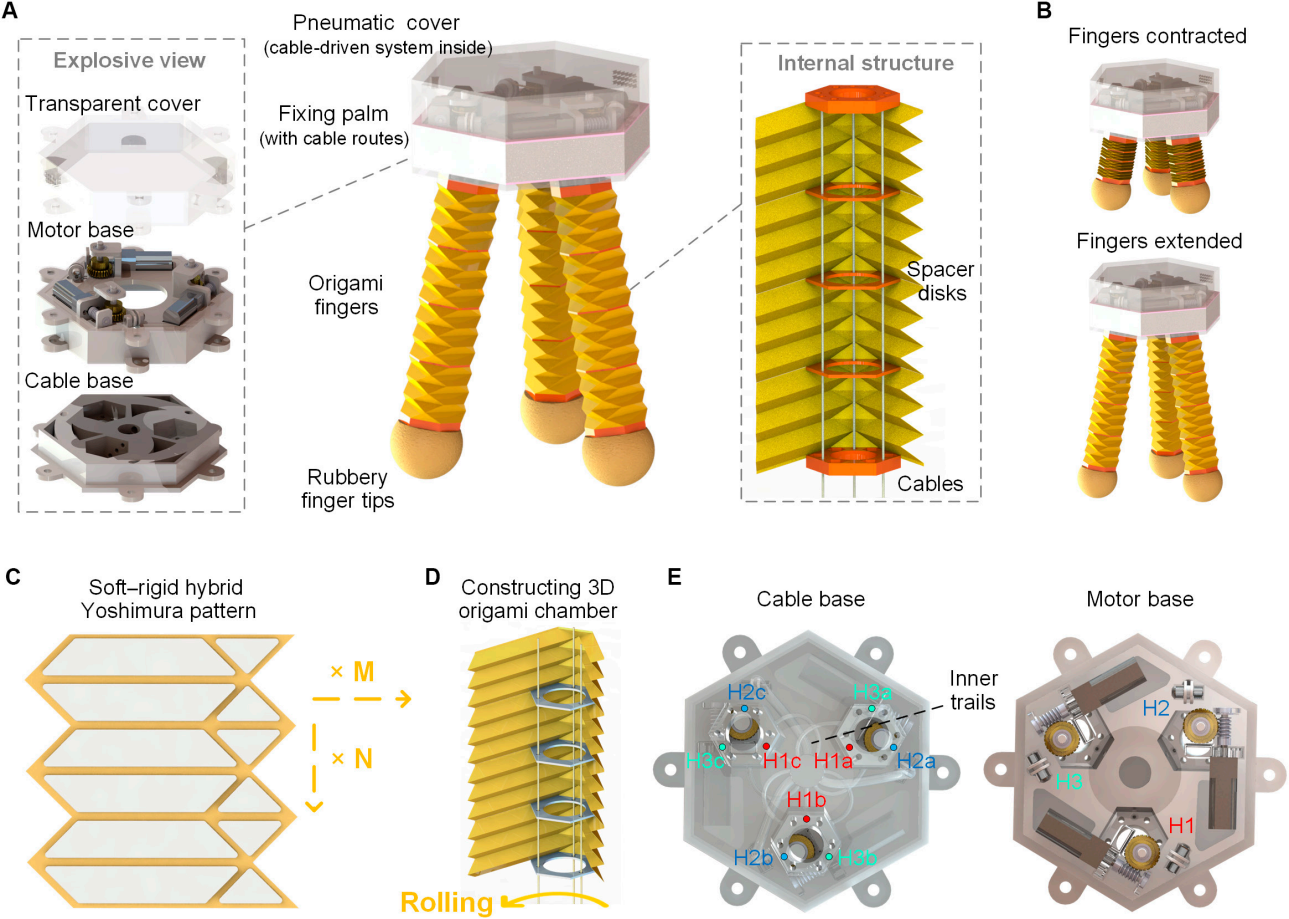
Construction of the origami gripper. (A) Structure of the gripper. (B) Tunable finger length. (C) The planar origami structure with a Yoshimura pattern. (D) The 3D Yoshimura chamber after rolling the planar origami structure. (E) The cable routes designed inside the fixing palm. Three motors are utilized to control the 9 cables inside 3 chambers, with each motor pulling 3 cables. Each 3 cables are joined together through the 3D printed routes.

The proposed gripper comprises 2 primary segments: the pneumatic origami chambers and the cable-driven system. As delineated in Fig. 2A, the origami chambers, namely, the finger structures, serves as the core function unit of the gripper, facilitating its interaction with various objects. Meanwhile, the cable-driven system, anchored to the fixing palm, predominantly governs the 3-DoF motion (contraction/extension and multidirectional bending) of the fingers. This origami chamber is characterized by its hollow, annular design, bridged between the fixing palm and a silicon finger tip at both its axial ends. Modular connections via bolts and nuts have been incorporated, permitting easy adjustments by altering chambers of different initial lengths for various requirements. Spacer disks are evenly distributed inside the chamber as showcased in Fig. 2A. Each of these disks embodies a hexagonal shape, with geometries of 1 mm in thickness and 15 mm per edge. Each disk is punctuated with 3 symmetrical holes, each measuring 1.2 mm in diameter. The trio cables in each chamber, navigating through all the disks, transmit the motion from the cable-driven system to the whole origami chamber. It is noteworthy that only 3 motors are utilized to drive the 9 cables inside the 3 chambers via 3 worm gears and 3 pulleys. Every 3 centrally symmetrical cables are controlled by the same motor through the fixing palm with predesigned, spatially arranged tendon routes (Fig. 2A). Consequently, the gripper is engineered to undergo contraction/extension when all motors are uniformly controlled (Fig. 2B) and to adopt an inward/outward bending when the motors are differentially controlled.

The origami chamber, as a crucial component of the length-tunable gripper to interact with the object, needs capabilities for bending and extension/contraction. To achieve this, a Yoshimura pattern [52] is employed in the construction of the chamber. This Yoshimura pattern forms a cylindrical origami structure that can be folded and bent while maintaining a certain level of torsional stiffness. Contrary to the prevalent use of low-stiffness sheets in creating Yoshimura cylindrical structures for applications as shells or springs [53–55], this prototype utilizes a combination of air-tight fabric and metal sheets to create a soft–rigid hybrid Yoshimura origami chamber. During the working process, the metal sheets serve as the rigid origami facets, whereas the soft fabric functions as the origami creases, facilitating shape morphing. The rigid metal sheets help maintain the structure of the chamber under internal positive pressure, preventing excessive deformation during the grasping process. The fabric, being nonstretchable, allows for easy inflation of the chamber with a relatively low input pressure. The incorporation of these mechanical features into the origami structure ensures a consistent folding process during the length adjustment and the grasping process. Moreover, the hexagonal Yoshimura structure, with its 3 convex and 3 concave sides, also provides an additional advantage. By placing the cables at the convex sides, interference between the cables and the chamber is minimized, ensuring smooth motion of the cables within the chamber.

As previously mentioned, the origami chamber is made from a soft yet nonstretchable material, and it naturally unfolds under internal positive pressure. Consequently, the primary mechanisms for achieving gripping and adjusting the finger length involve winding and releasing the 3 cables. However, the role of pneumatic actuation in this system is still crucial. Given that the cables and the origami chamber are susceptible to passive compression and bending, they struggle to maintain the finger shape on their own. This is where positive pressure comes into play, inflating the sealed origami chamber to provide support and maintain the overall structure. The dual actuation sources cable-pulling and air-pushing work antagonistically and ensure that the cables are in tension. Altering the input pressure further results in changes to the internal forces, allowing for adjustments in the stiffness of the origami chamber. Thus, this hybrid and redundant actuation system enables a degree of active control over the finger’s movements and its stiffness. The ability to adjust stiffness is a crucial attribute for soft grippers, as it significantly amplifies their load-carrying capabilities [56]. Therefore, the design that we propose synchronously ensures the feasibility of length adjustment and stiffness modification, thereby enhancing the gripper’s versatility and adaptability.

### Fabrication and prototyping

The soft–rigid origami chamber can be fabricated using a layer-based method, similar to the approach we outlined in our previous work [17]. To enhance the air tightness and facilitate self-adhesion, the soft fabric is coated on both sides with TPU. As depicted in Fig. 2C, 2 varieties of 0.3-mm-thick metal sheets are strategically attached to the fabric, following the 2-dimensional Yoshimura pattern, and are spaced 2 mm apart from each other. Subsequently, by wrapping this 2-dimensional hybrid structure around 2 end plates (namely, the fixed and moving plates of the origami chamber) and the spacer disks, a 3-dimensional (3D) chamber boasting a cylindrical origami structure is achieved, as shown in Fig. 2D. During the fabrication process, we firstly connect laser-cut metal sheets to the fabric base with glue, then wounding the fabric to form a closed, cylindrical structure. The area where the fabric overlaps is sealed via welding with a 180 °C electric soldering iron. Later, we bolt the tip of the finger with an end cap. All the end plates and spacer disks are crafted from 3D printed resin parts (8000, Somos) and can be securely bonded with the TPU-coated fabric using adhesive (5569, Kaibingtuan). The spacer disks are further treated with a polytetrafluoroethylene coating spray (571, OKS), substantially reducing friction between the small holes and the cables. Furthermore, we have opted for 0.5-mm polytetrafluoroethylene-coated fiberglass threads as our choice of cables. Given that these cables need to be pulled out from the pneumatic chamber, silicone seal pads (DragonSkin20, Smooth-On) are fabricated and integrated between the fixing palm and each finger. To minimize friction of the cables during the pulling process, grease is applied within the holes of silicone pads and along the trails inside the fixing palm. It is noteworthy that the fabricated origami finger performs good robustness, which can withstand a maximum pressure of more than 30 kPa. The air tightness is not substantially changed after 1,000 times of inflating and deflating during the testing process.

As to the control parts, the pneumatic pressure is precisely regulated by an off-board proportional valve (ITV1010, SMC), paired with a pressure sensor (PSE532, SMC). The on-board micromotors and the proportional valve are simultaneously controlled through pulse-width modulation signals, which are generated by an off-board microcontroller (Mega 2560, Arduino). A digital-to-analog converter module (DC2376A, ADI) is connected in between the controller and the proportional valve, ensuring a stable voltage input is maintained. This configuration allows for the establishment of a closed-loop system, capable of maintaining the inner pressure of the chamber at a near-constant level throughout its motion. The micromotors, equipped with Hall encoders, facilitate precise control over the position and velocity of the tendon-driven system, thanks to a cascaded feedback proportional-integral controller. To guide the tendons with minimal friction before they are wound, 3 nylon roller wheels are employed as pulleys. Finally, the prototype is assembled by integrating the manually crafted origami chamber, dc motors, worm gears, roller wheels, and other standard components (such as bearings, bolts, and nuts) onto the 3D printed fixing palm (8000, Somos) and are sealed with a 3D printed transparent cover (WaterClear, Somos) and customized silicone seal pads.

### Modeling of the origami finger

**Fig. 3.**
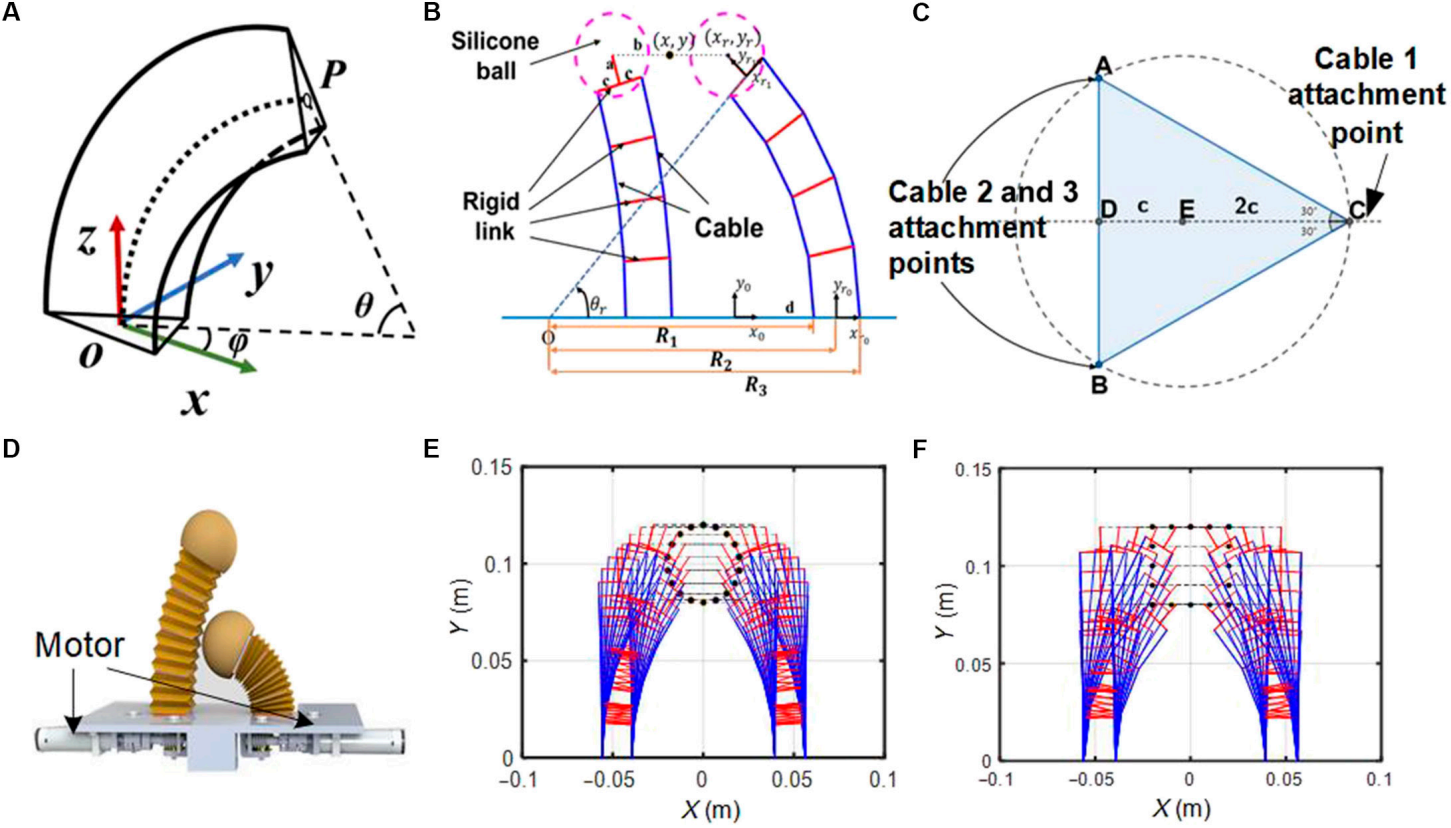
Schematics of the gripper. (A) The equivalent model of the cable-driven finger. (B) A 2-fingered prototype for in-hand manipulation. (C) The locations of the cables of a specific chamber. (D) The schematic diagram of the 2-fingered gripper. (E) The calculation results for the gripper to achieve a circular trajectory. (F) The calculation results for the gripper to achieve a square trajectory.

Among many modeling methods of soft robots, the constant curvature assumption has been widely used due to its simplicity and accuracy. This study shows that the constant curvature assumption is well applied to the modeling and analysis of our proposed origami finger. As shown in Fig. 3A and B, the cross-section of our proposed origami finger can be approximated by equilateral triangles. Among them, the vertex of the triangle indicates the position of the pull cable. When the length of the cable changes, the finger will bend from the initial vertical state, and the deformation of its axis can be approximated as a circle with constant curvature. In this case, the position P of the finger axis at the end can be represented by 3 basic variables, namely, the radius of curvature *R*, the center angle *θ*, and the rotation angle *φ* of the arc center line in the *xy* plane. Here, we use [*x_P_*, *y_P_*, *z_P_*] to represent the coordinates of the end of the finger bending axis, so the basic variable of the constant curvature law can be expressed as follows:

$$\left\{\begin{array}{c} \varphi =\text{arctan}\left(\frac{y_{P}}{x_{P}}\right)\\ R=\frac{x_{P}^{2}+y_{P}^{2}+z_{P}^{2}}{2\sqrt{x_{P}^{2}+y_{P}^{2}}}\\ \theta =\arccos\left(1-\frac{\sqrt{x_{P}^{2}+y_{P}^{2}}}{R}\right) \end{array}\right.$$ (1)

We further develop an inverse kinematic model of this finger. In the constant curvature model assumption, the shape of the equivalent section of the finger at any position is the same equilateral triangle, which means that the curvature of the 3 cables is always the same. As shown in the following equation, the corresponding kinematic model of the finger can be established by calculating the arc length of each cable:

$$C_{i}=2n\;\sin\frac{\theta }{2n}\left[R-\text{dcos}\left(\frac{2\pi }{3}\left(i-1\right)+\frac{\pi }{2}-\varphi \right)\right]$$ (2)

where *C_i_* = [*C*_1_, *C*_2_, *C*_3_] are the lengths of the 3 cables.

### Modeling of the 2-finger gripper

To demonstrate the in-hand manipulation capacity of the proposed gripper design, we built a 2-finger gripper to perform the planar manipulation task, considering the complexity of the control systems of the 3-fingered manipulation. As shown in Fig. 3D, 2 identical fingers are arranged in parallel on the base platform. Two motors at the bottom of the base are used to change the length of the cable inside the chamber. The planar manipulation task only requires 2 cables per finger to control the motion of the finger tip. Based on the finger kinematics model proposed in the last section, we can establish the single-finger kinematics model of the 2-finger gripper:

$$\left\{\begin{array}{c} R_{x}=\frac{x_{1}^{2}+y_{1}^{2}}{2x}\\ \theta_{r}=\arccos\frac{R_{x}-x_{1}}{R_{x}}\\ x_{r}=x_{1}-a\;\sin\;\theta_{r}\\ y_{r}=y_{1}+a\;\cos\;\theta_{r} \end{array}\right.$$ (3)

where *R_x_* represents the *x* coordinate value of *O* in the frame {*o*_*r*_0__, *x*_*r*_0__, *y*_*r*_0__}, (*x*_1_, *y*_1_) represents the coordinates of the origin of frame {*o*_*r*_1__, *x*_*r*_1__, *y*_*r*_1__} in frame {*o*_*r*_0__, *x*_*r*_0__, *y*_*r*_0__} and *a* represents the distance from the center of the finger tip to the top platform of the inflatable chamber. In addition, it is preset that the center of both fingers and the object are at the same height. Thus, the following equation can be obtained:

$$\left\{\begin{array}{c} x_{r}=x+b-d\\ y_{r}=y \end{array}\right.$$ (4)

where *b* represents the half of the sum of the side length of the object to be grabbed and the diameter of the silicone ball, *d* is the distance between the origin of frame {*o*_*r*_1__, *x*_*r*_1__, *y*_*r*_1__} and frame {*o*_*r*_0__, *x*_*r*_0__, *y*_*r*_0__}, and (*x*, *y*) is the position of the grabbed object in frame {*o*_*r*_0__, *x*_*r*_0__, *y*_*r*_0__}. According to Eqs. 3 and 4, *R_x_* and *θ_r_* can be calculated for any given position of the object (*x*, *y*) on the premise that the size of the object is known.

According to Fig. 3B and C, the wiring in the right finger is presented. Therefore, the radius of the circle of constant curvature (*R*_1_) where the 4 nodes of cable 2 are located differently *a* from that of the central axis circle of constant curvature (*R*_2_). The relationship between the 3 radii can be expressed as ∣*R*_1_ − *R*_2_∣  = *c* and ∣*R*_3_ − *R*_2_∣  = 2*c*, where c = 5.5 mm and *R*_2_ =  ∣*R_x_*∣, when *R_x_* < 0, *R*_1_ < *R*_2_ < *R*_3_; otherwise, *R*_1_ > *R*_2_ > *R*_3_. According to the constant curvature hypothesis, the following expressions can be obtained:

$$\left\{\begin{array}{c} L_{\mathrm{rl}}=2nR_{1}\;\sin\;\frac{\theta_{r}}{2n}\\ L_{\mathrm{rr}}=2nR_{3}\;\sin\;\frac{\theta_{r}}{2n} \end{array}\right.$$ (5)

where *n* (=3) is the number of segments, *L_rl_* and *L_rr_* represent the length of the cable on the left (cable 2 and cable 3) and right (cable 1) of the axis of the right finger.

Due to the axial symmetry of left and right fingers, the inverse kinematics equation of left fingers can be also listed and solved. After the completion of the above work, the inverse solution can be carried out according to the movement trajectory of the captured object. We validate the model through 2 sets of simulation experiments. In order to simulate the grasping of an object, a set of circular target trajectories and a set of square target trajectories are designed respectively. The motion trajectories of the 2 grippers are solved by the established inverse kinematics. As shown in Fig. 3E and F, our kinematic model can accurately solve the motion trajectory of the whole gripper.

## Results and Discussion

### Gripping test with tuning of finger length and stiffness

The designed origami gripper mainly exhibits 2 functionalities: variable finger lengths and variable stiffness. Therefore, when grasping various objects, it can actively adjust the effective length of the fingers controlled by motors and the structural stiffness of the fingers regulated by pneumatic pressure, according to the volume and weight of the object. To validate the improvement in grasping performance, we first conducted grasping force tests on a gripper prototype.

**Fig. 4.**
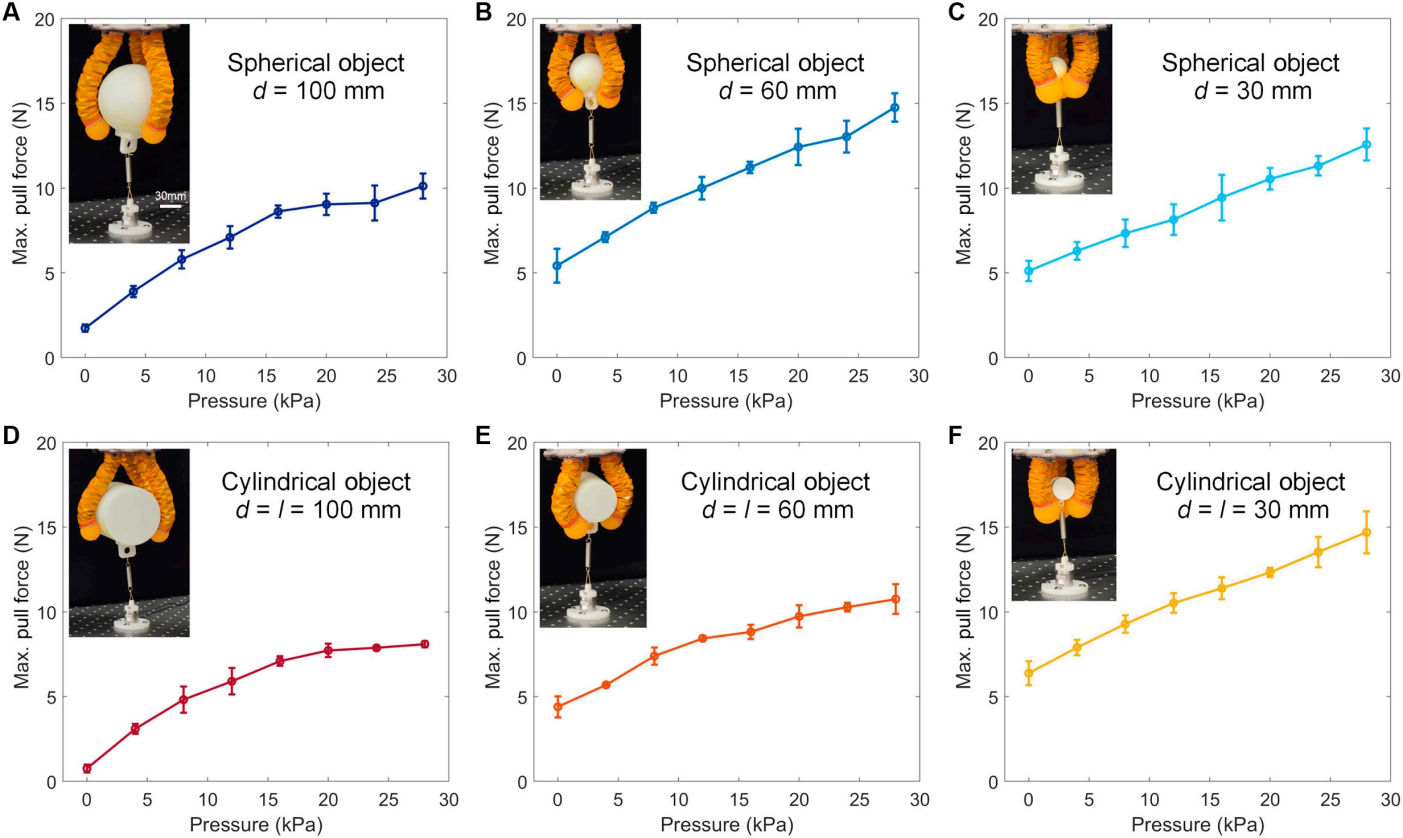
Variable stiffness tests of the origami gripper under different finger lengths. (A to C) Pull-off force of the gripper when a grasping spherical objects with diameters from 100 to 30 mm, under various input pressure values. (D to F) Pull-off force of the gripper when a grasping cylindrical objects with diameters and axial lengths from 100 to 30 mm, under various input pressure values.

As illustrated in Fig. 4, the experimental setup consists of customized grasping objects, a spring, and a force sensor (Nano 25, ATI Inc.). The customized grasping objects are 3D printed parts in 3 different sizes, both spherical and cylindrical in shape, connected to the force sensor fixed on a stable platform via a tension spring. During the experiment, the origami gripper is fixed upon the end effector of a 6-DoF industrial robot (UR10, Universal Robots Inc.) and initially envelops the customized grasping object to be grasped in a compliant state with an initial input pressure of 5 kPa. We then adjusts its internal pressure to a set value, followed by vertically lifting the gripper through the industrial robot until the customized grasping object is released from the gripper. Each experimental trial is conducted 5 times to reduce error fluctuations. Subsequently, the maximum grasping force corresponding to different states (both finger length and stiffness) can be extracted from the tensile force data recorded by the force sensor throughout the entire process.

Figure 4A to C displays the grasping performance of the origami gripper for spherical objects with diameters of 100, 60, and 30 mm. According to the experimental images, it is evident that the finger length of the gripper changes substantially according to the size of the spherical objects, keeping an enveloping grasp of objects with varying dimensions. In contrast, if the finger length were nonadjustable, using the finger length from the 30-mm-diameter spherical test as the fixed length for the gripper would render it incapable of grasping objects with diameters of 60 and 100 mm. Similarly, if the finger length from the 100-mm-diameter spherical test were used as the fixed length, the gripper would struggle to stably grasp objects with diameters of 30 and 60 mm due to the excessive length and extra space. Figure 4D to F presents similar experimental scenarios using cylindrical grasping objects. Hence, the fingers capable of adjusting their effective lengths substantially enhance the applicability of the gripper. Meanwhile, the modeling of the hybrid driven origami structure will also provide theoretical guidelines for determining suitable grasping shapes of the fingers.

In addition, Fig. 4 also depict the variations in maximum grasping force resulting from tuning the internal pressure of the gripper under different grasping states. Throughout the experiment, the pressure was varied within a range of 0 to 28 kPa. The experimental results clearly indicate that the grasping force of the gripper increases substantially with an increase in internal pressure, suggesting that variations in pressure can substantially alter the structural stiffness of the flexible origami structure. As a result, an enhancement in its load-bearing capacity can be achieved through an increase in input pressure. When the finger length is shorter, the maximum grasping force exhibits an approximate linear relationship with the input pressure. The alteration in the shape of the grasping object does not substantially impact its grasping capabilities. In contrast, when the finger length is longer, the maximum grasping force demonstrates an approximate linear relationship with the input pressure at lower pressures, while at higher pressures, the increase in stiffness occurs at a relatively slower pace. This phenomenon is likely attributed to the increase in the moment arm resulting from the extended finger length.

### Grasping performance

To verify the practical grasping capability of the designed origami gripper, we further conduct grasping tests on selected daily and laboratory items based on the Yale-Columbia-Berkeley Object Set (YCB object set) [57]. The items cover a wide range of sizes and weights, including small and lightweight objects like ping-pong balls, fabric pieces that conform to the desktop, thin bats, as well as heavier objects like copper wire coils, and larger and heavier objects like football. The grasping process is divided into the following 4 steps:

1. Move directly above the target object, and determine the finger length and initial grasping posture based on the shape and volume of the object;

2. Move vertically downward to make full contact with the target object;

3. Perform grasping movement by controlling the cables inside the fingers, and adjust the pneumatic pressure of the fingers based on the weight of the object after the grasp is completed;

4. Move vertically upward and remain stationary in mid-air for 5 s to ensure effective grasping of the object.

**Fig. 5.**
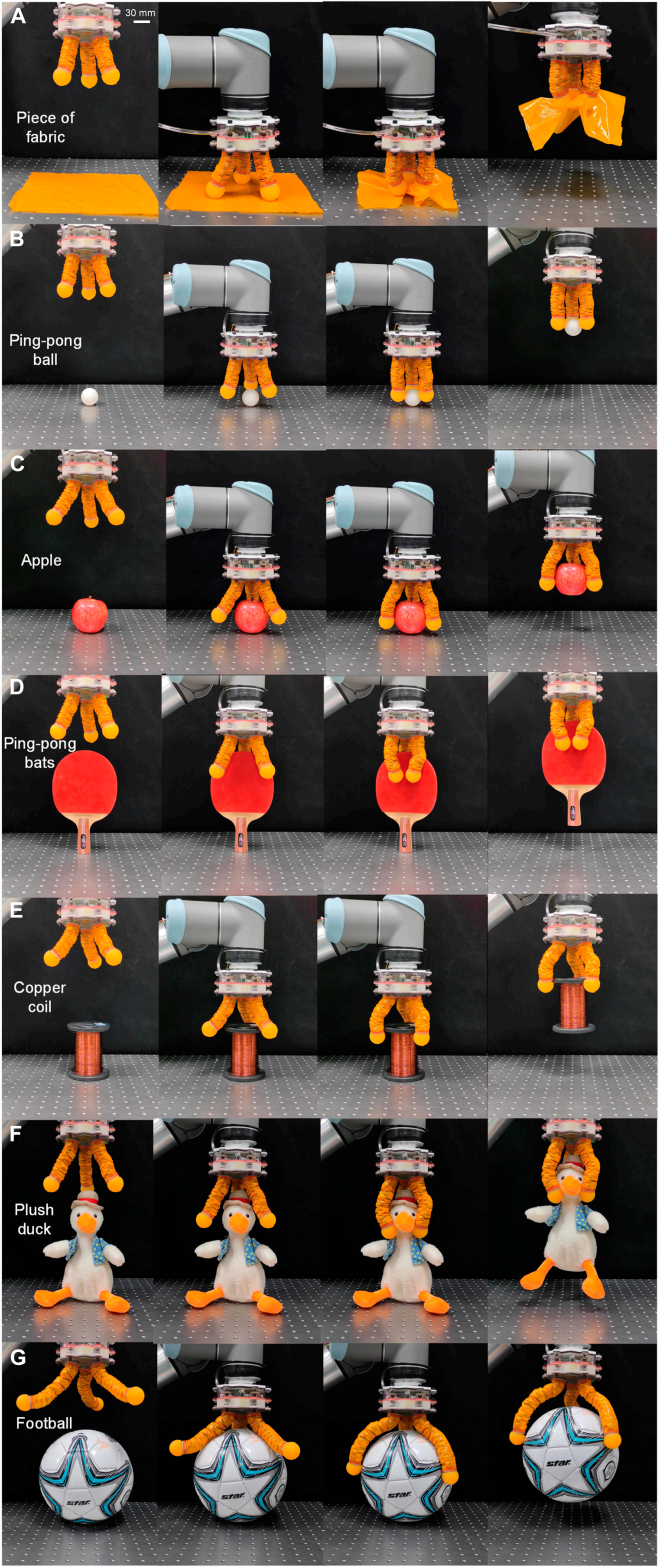
Grasping tests of different objects. (A to G) Grasping of a piece of fabric, a ping-pong ball, an apple, a ping-pong bat, a copper coil, a plush duck, and a football.

Figure 5 demonstrates the grasping process and results for some of the tested items. Thanks to the advantage of tunable finger lengths, the origami gripper successfully envelops and grasps spherical objects of different volumes, from a ping-pong ball to a football. Moreover, due to the structural compliance of the gripper, it can conform to a planar desktop, enabling the effective grasping of soft and thin fabric laid flat on the table. At the same time, the structural adaptability of the origami structure allows the 3-fingered gripper to grasp irregularly shaped objects, such as a flat ping-pong bat. Additionally, benefiting from its capability of stiffness tuning, this soft gripper can also effectively grasp heavy objects, such as a copper wire coil weighing nearly 1 kg. Furthermore, since the control of the grasping process (cable-driven) and the variable stiffness (pneumatic-driven) are intrinsically decoupled, the gripper can also achieve a soft enveloping grasp on softer objects like a plush duck. After forming the enveloping grasp, the finger stiffness can be enhanced via increasing the input pressure, ensuring effective grasping without causing damage to the object.

### In-hand manipulation

**Fig. 6.**
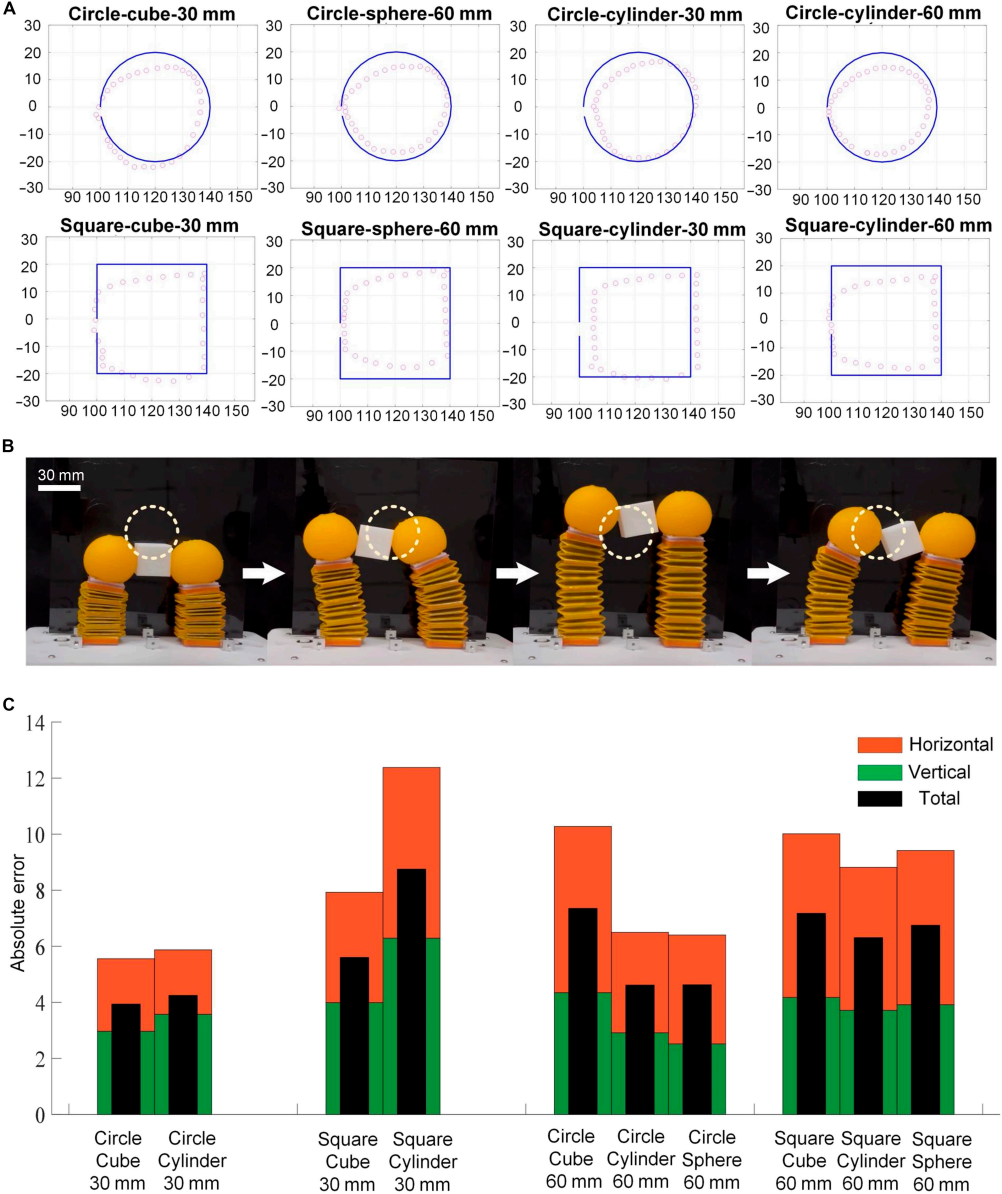
In-hand manipulation tests. (A) Manipulating different objects (cube, sphere, and cylinder with diameter/side length of 30 and 60 mm) to follow different trajectories (circle and square). The solid lines denote idea trajectories. The dots are measured positions of the centers of different objects. (B) Manipulation process of carrying a cube to follow a circle trajectory. (C) Absolute positioning accuracy of different manipulation tests.

To verify the in-hand manipulation capability of the designed origami gripper, benefiting from the controllable finger motions, the tunable finger lengths, and the constructed theoretical model, various path-following tests were conducted with 4 different manipulated objects and 2 kinds of trajectories. The 4 objects include a cube with 30-mm side lengths, a sphere with a 60-mm diameter, and 2 cylinders with diameters of 30 and 60 mm. The 2 trajectories are a circle with a 40-mm diameter and a square with 40-mm side lengths. As shown in Fig. 6A, all 4 objects can be manipulated by the gripper to follow the 2 predesigned trajectories. Although small deviations still exist between the real motions and the ideal ones, the manipulation function is fully validated, especially considering its soft structure. Figure 6B illustrates the manipulation process of a specific test, namely, carrying a cube with 30-mm side lengths to follow a circle trajectory. Apparently, this soft gripper can also move the object in addition to grasping, without requiring an additional robotic arm, further improving the functionalities of soft grippers. In addition, according to Fig. 6A, it seems that the shape and the size of the objects do not show a substantial influence on the manipulation performance. Therefore, we further extracted the positioning errors of the measured trajectories to study the effect of the object shape and size on the manipulation performance. Figure 6C illustrates the absolute positioning error of the end in the horizontal and vertical directions and the total displacement, respectively. The results show that the error of manipulation in the vertical direction is larger than the horizontal direction. In addition, the gripper performs relatively better under circle trajectories and with smaller object, while the shape does not obviously affect the in-hand manipulation. As delineated in Table, our proposed gripper, when juxtaposed with extant studies, exhibits better in-hand manipulation capacity while concurrently performing high grasping force and range. Thus, this design holds the promise of expanding the utility of robotic fingers for better compliant grasping and manipulation.

**Table.** Performance comparison among grippers

Gripper type	Grasp range	In-hand manipulation ability	Max. grasp force
Hybrid [50]	10–80 mm	N	1.2 N
Hybrid [57]	10–325 mm	N	30 N
Hybrid [37]	10–60 mm	N	8 N
Soft [28]	N/A	N	61 N
Soft [58]	3–150 mm	N	40 N
Jamming-based [59]	2–90 mm	N	8 N
Hybrid (this work)	20–130 mm	Y	15 N

N/A, not applicable.

## Conclusion

In this study, we introduce a soft gripper inspired by the retractable tentacle of the cuttlefish. By integrating the fabric-based soft–rigid origami pneumatic chamber with motor-driven cables in an antagonistic arrangement, the gripper exhibits the unique ability to adjust finger stiffness upon variation of internal pressure and finger length upon controlling the cable lengths. Additionally, precise control over cable length facilitates the motion control of the finger tips.

To accurately predict the trajectory of the gripper, we establish an inverse kinematics model for the fingers. Furthermore, we conduct experiments to assess the stiffness during the grasping process under different finger lengths. Results demonstrate that the gripper’s maximum grasping force increases proportionally with internal pressure. The variability in finger length also expands the gripper’s grasp range. In practical tests, the gripper exhibits remarkable versatility, successfully grasping objects ranging from thin fabric pieces (0.1-mm thickness) to larger items such as a football (diameter 400 mm) with a maximum grasping force exceeding 15 N. The gripper’s adaptability is further showcased in in-hand manipulations, where a 2-fingered gripper efficiently tracks objects of diverse shapes and sizes. The maximum positioning error observed in path tracking tests is 8 mm, demonstrating the gripper’s potential for precise interactions with targets. While exhibiting great potential, current limitations of the gripper include a manual manufacturing process of the origami chamber and the absence of sensing capabilities. Future efforts will focus on optimizing the fabrication process and incorporating sensors into fingers, enhancing its capabilities for more sophisticated human–robot interactions.

## Data Availability

All data needed to evaluate the conclusions in the paper are present in the paper and/or the Supplementary Materials. Additional data related to this paper may be requested from the authors.
